# Evaluation of antibacterial and antifungal properties of a tissue conditioner used in complete dentures after incorporation of ZnO‒Ag nanoparticles

**DOI:** 10.15171/joddd.2019.002

**Published:** 2019-04-24

**Authors:** Seyed Amin Mousavi, Reza Ghotaslou, Abolfazl Akbarzadeh, Niloufar Azima, Ali Aeinfar, Azin Khorramdel

**Affiliations:** ^1^Department of Prosthodontics, Faculty of Dentistry, Tabriz University of Medical Sciences, Tabriz, Iran; ^2^Department of Medical Microbiology, Faculty of Medical, Tabriz University of Medical Sciences, Tabriz, Iran; ^3^Department of Medical Nanotechnology, Faculty of Advanced Medical Sciences, Tabriz University of Medical Sciences, Tabriz, Iran; ^4^Department of Pediatric, Faculty of Dentistry, Tabriz University of Medical Sciences, Tabriz, Iran; ^5^Private Practice, Tabriz, Iran; ^6^Department of Periodontics, Faculty of Dentistry, Tabriz University of Medical Sciences, Tabriz, Iran

**Keywords:** Antibacterial, antifungal, tissue conditioner, ZnO‒Ag nanoparticles

## Abstract

***Background***. Incorporation of antifungal and antimicrobial agents into tissue conditioners might inhibit the formation of microbial plaque and prevent complications such as denture stomatitis. The present study was carried out to evaluate the antibacterial and antifungal properties of a tissue conditioner after incorporation of ZnO‒Ag nanoparticles into their structure.

***Methods***. In this in vitro study, 4 microorganisms were evaluated at 6 concentrations of ZnO‒Ag nanoparticles at 24- and 48-hour intervals, using 168 samples. The nanoparticles were mixed at a ratio of 50% Ag and 50% ZnO and were homogenized with the tissue conditioner at 0.625, 1.25, 2.5, 5, 10 and 20 wt% according to the MIC technique principles. After culturing the microorganisms, a spectrophotometer was used for determining proliferation of microorganisms with the use of turbidity after 24 and 48 hours of incubation at 37ºC.

***Results***. Complete inhibition of the proliferation of *Candida albicans, Enterococcus faecalis *and *Pseudomonas aeruginosa* was observed at 24- and 48-hour intervals at a concentration of 10%; such inhibition was observed at 20% concentration of nanoparticles with *Streptococcus mutans*. In addition, the most effective concentration of ZnO‒Ag nanoparticles at both 24- and 48-hour intervals was 5% with *C. albicans* and 2.5% with *E. faecalis*. In addition, the most effective concentration at 24- hour interval with *S. mutans* was 10% and with *P. aeruginosa* they were 5% at 24-hour and 2.5% at 48-hour intervals.

***Conclusion***. Incorporation of ZnO‒Ag nanoparticles into tissue conditioners resulted in the inhibition of bacterial proliferation

## Introduction

Tissue conditioners are soft and flexible materials that are used to treat inflammation and tissue injuries of the oral cavity and to take functional impressions. These materials are usually used as a temporary relining material, in the tissue repair stage after placing implants and in maxillofacial dentures.^1^

These materials exhibit time-dependent cushioning effect and help in the healing of traumatized tissues and in the retention of intraoral and extraoral removable prostheses.

They are also used for palliative and diagnostic purposes, for restoring the vertical dimension of occlusion and for correcting the occlusal relationships of old prostheses.^2^ Unfortunately, in some cases these tissue conditioners provide a proper environment for the proliferation and colonization of different microorganisms, which might aggravate the complications due to the use of dentures.^3,4^

One of the most common complications of wearing complete dentures is denture stomatitis or atrophic chronic candidiasis. With continuous use of dentures, the tissue side of the denture and the space created between the tissue surface and the mucous tissue of the patient gradually becomes susceptible to the growth and colonization of various microorganisms.^5^

Several techniques have been suggested to prevent accumulation of plaque and proliferation of fungi, including *Candida* species, on tissue conditioners, which include incorporation of different kinds of antifungal agents into tissue conditioners,^5-10^ use of varnishes containing antifungal agents^11-13^ or immersion of the denture relined with tissue conditioners in antiseptic solutions.^14^

Incorporation of antifungal agents into tissue conditioners might be a hypothetical solution for this therapeutic problem. The main advantages of incorporating antifungal agents into tissue conditioners might include a decrease in cost, no need for cooperation of patients, simultaneous treatment of *Candida* infections and the injured mucosa under the denture, and a decrease in the procedural steps.^5^

Silver ions and its salt derivatives have strong antibacterial activity, in addition to advantages such as low toxicity, proper biocompatibility with human cells, long-term antibacterial activity due to the release of ions^15,16^ and development of no bacterial resistance against them.^17^

In an in vitro study, Nam et al (2011) incorporated 0.5 wt% of silver nanoparticles into Soft-Liner (GC) tissue conditioner and prepared disk samples, reporting complete inhibition of colonization of fungal species, including *Candida*. They inhibited colonization of *S. mutans* and *S. aureus* in that study by incorporating 0.1 wt% of these nanoparticles into the tissue conditioner.^18^ In addition, it was demonstrated that there was inhibition of bacterial growth in test tubes even at low concentrations of silver nanoparticles; however, it was possible to increase the duration of the release of silver ions and the duration of antibacterial properties by increasing the concentration of silver nanoparticles before it reached the toxic level.^18^

Liz et al (2014) reported that silver nanoparticles incorporated into acrylic resin at >1‒5 wt% exhibited antifungal activity and inhibited formation of biofilms.^19^ Kristinakairyte (2014) showed that the suspension of ZnO nanoparticles exhibited antifungal and antimicrobial activity.^20^ Although various studies have evaluated the antimicrobial properties of silver and zinc oxide, no studies to date have evaluated the combined incorporation of these nanoparticles into tissue conditioners in relation to their antibacterial and antifungal effects. The present study was undertaken to evaluate the antibacterial and antifungal properties resulting from the incorporation of ZnO‒Ag nanoparticles into a tissue conditioner. Due to a lack of data on the subject, the wt% in the present study consisted of a wide range, including 0.625%, 1.25%, 2.5%, 5%, 10% and 20% in order to determine the minimum percentage necessary for inhibiting the microorganisms.

## Methods

The bacterial species used for microbial tests consisted of *S. aureus* (ATCC6538), *P. aeruginosa* (ATCC9027) and *E. faecalis* (ATCC29212), and C. albicans (ATCC10231) fungal species, which were provided by the Microbiology Laboratory of Tabriz Faculty of Medicine. Brain-heart broth (Merck, Darmstadt, Germany) and blood agar culture media were used to proliferate the bacterial species.

### Synthesis of ZnO‒Ag Nanoparticles

The ZnO‒Ag nanoparticles were synthesized using the photo-precipitation technique. In this technique, a certain amount of zinc acetate was dissolved in ethanol and distilled water. Then an ultrasound device was used for homogeneous dispersion of the nanoparticles, followed by heating at 80ºC for 2 hours in a reflux reactor. Subsequently, a certain amount of silver nitrate salt was added to the warm solution and the reflux continued for one hour. The resultant solution was agitated without heating for 2 hours under ultraviolet light. Finally, the resultant precipitate was centrifuged and transferred into an oven and calcinated at 550ºC. The final product was ZnO‒Ag.

The particles of the materials used consisted of the following:

ZnO (purity: 99%; size: 10‒30 nm; nearly spherical)
Ag (purity: 99‒99%; size: 20 nm; spherical).
The produced ZnO‒Ag nanoparticles were verified using SEM images, x-ray diffraction technique and FT-IR technique.

### Incorporation of ZnO‒Ag Nanoparticles into the Tissue Conditioner

The prepared nanoparticles were mixed together at 50% Ag and 50% ZnO ratios, with wt% of 0.625, 1.25, 2.5, 5, 10 and 20. They were homogenized with the tissue conditioner based on the principles of MIC (minimum inhibitory concentration). The tissue conditioner used was a product of GC.

It should be pointed out that in determining the concentrations of nanoparticles attention was paid to the fact that incorporation of nanoparticles at concentrations >20% results in toxic effects.^18^ Therefore, the highest concentration of nanoparticles in the present study was 20%. Then based on the principles of the MIC technique, this value was halved for dilution in each stage.^21^ The reason for mixing with fluid was better homogenization and preparation of a more homogeneous solution. The prepared mix was placed in acrylic molds measuring 2×6×8 mm.^18^ The molds were fabricated in the laboratory using self-cured acrylic resin used to fabricate the base for complete dentures; then cubes with the above-mentioned dimensions were prepared within the acrylic resin masses. The depth or in fact the thickness of the prepared cube was 2 mm.

### Culturing of Microorganisms

The MIC technique was used to determine the concentration of nanoparticles that inhibited bacterial growth. MIC is defined as the minimum concentration of antimicrobial agents that clearly inhibits the growth of microorganisms.^22^

In the present study, 4 bacterial and fungal species, representing various pathogenic agents, were used as follows:^23^

- *S. aureus* (ATCC6538) as a representative of gram-positive and non-sporogenic bacteria
- *P. aeruginosa* (ATCC9027) as a representative of resistant gram-negative bacteria
- *C. albicans* (ATCC10231) as a representative of pathogenic fungal species in the oral cavity
- *E. faecalis* (ATCC29212) as a representative of resistant gram-positive bacteria

Brain-heart broth (Merck, Darmstadt, Germany) was prepared at a concentration of 30 g/lit according to manufacturer’s instructions for proliferation of the microorganisms under study and then sterilized in an autoclave.^18^ Blood agar culture medium, too, was prepared using the conventional technique for culturing the samples. To this end, Mueller-Hinton broth medium was prepared, sterilized in an autoclave and cooled down to room temperature. Then sheep blood was added to it up to 17 vol% and placed in sterile 8-cm plates for culturing the samples.

The fungal or microbial strain that was prepared in lyophilized powder form was dissolved in the brain-heart broth medium and cultured on sterile plates after incubation for 24 hours at 37ºC to grow the microorganisms. Then 0.5% McFarland suspension, i.e. 1.5 ×10^8^ CFUs, was prepared from the grown fungi. Then the suspension was diluted to achieve a concentration of 1.5 ×10^5^ CFU.^18^

To prepare the fungal suspension at a concentration of 1.5×10^5^ CFU, 1 mL of the suspension at a concentration of 1.5 ×10^8^ was mixed with 9 mL of the culture medium and a suspension was achieved with a concentration of 1.5 ×10^7^, and this was repeated twice to achieve a suspension with a concentration of 1.5 ×10^5^. A sampler was used to transfer 1.5 ×10^5^ CFU of the final suspension into each test tube and then ZnO‒Ag nanoparticles were added to the tissue conditioner, followed by incubation at 37ºC for 24 and 48 hours.^18^

### Determining Bacterial Growth by Measuring Turbidity

A spectrophotometer (EPOCH, Bio Tek Instruments Inc, Highland Park) was used to determine the growth of microorganisms based on turbidity. The readings were carried out at a wavelength of 600 nm.

### Analysis of Data

The sample size was calculated at 6 samples in each study group by considering α=0.05, a study power of 80%, a standard deviation of 0.05 for the control group, a standard deviation of 0.2 for the study groups and an expected difference of 2 units. Since there were 4 microorganisms and 7 concentrations (including the control tube) a total of 28 groups (168 samples) were evaluated. The results of turbidity testing were analyzed with SPSS 20. ANOVA was used to compare the growth of microorganisms at different concentrations of nanoparticles; post hoc Tukey tests were used for two-by-two comparisons of the groups.

## Results

### Evaluation of SEM Images

**Figure 1:**
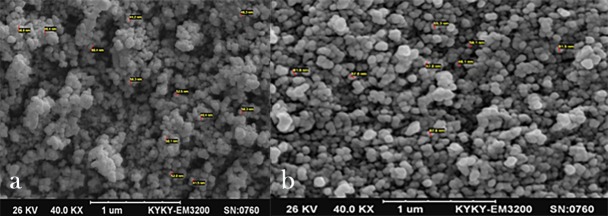
SEM images of Ag (a) and ZnO (b) nanoparticles.

The nanoparticles were dissolved in a small amount of water and the resultant suspension underwent SEM imaging on a gold grid. Figure 1 shows SEM images of the prepared nanoparticles. The electron microscope images of nanoparticles were prepared using the SEM technology. After precise and point-by-point evaluation of different parts of the samples, the sizes of magnetic nanoparticles were determined at 40‒100 nm.

### Evaluation of X-ray Diffraction (XRD)

**Figure 2:**
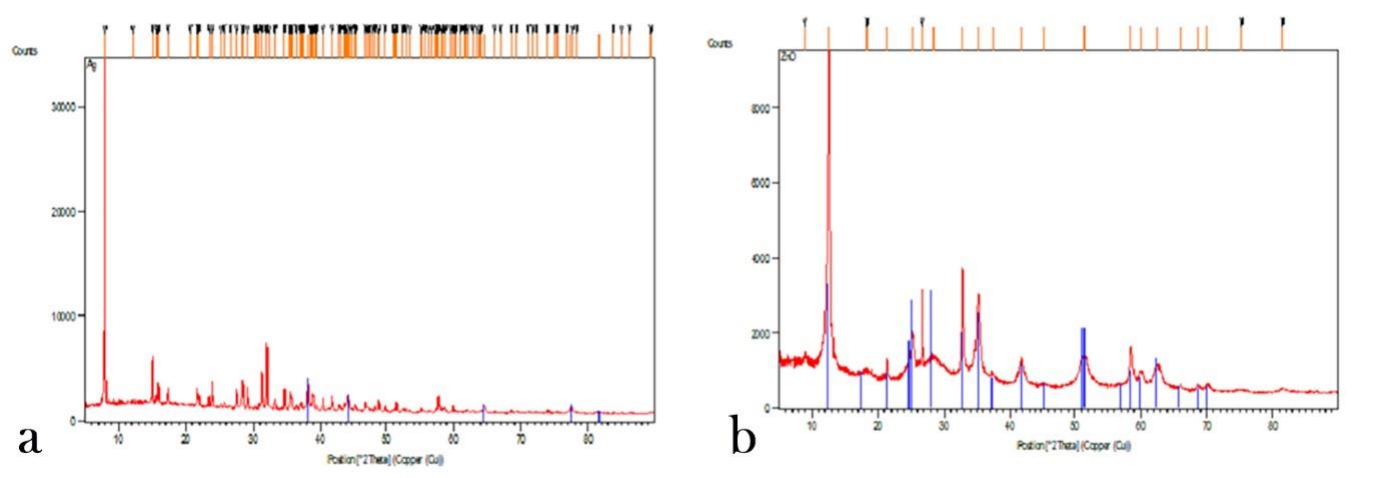
XRD images of Ag (a) and ZnO (b) nanoparticles.

The crystalline structure of the synthesized magnetite was analyzed with the use of XRD technique. 2θ value was considered at a range of 20‒100. As expected, in the images acquired (Figure 2), the silver nanoparticles (a) and zinc oxide nanoparticles (b) corresponded to the spectrum lines in the references. In crystals of nanoparticles, the highest peaks indicated the mean size of the crystals and the Debye-Scherrer formula was used to determine the nanoparticle range size:

D_hkl_=0.9λ(βcosθ)

in which β indicates half of the width of x-ray diffraction (XRD) and λ=0.154 nm and θ is the half of the diffraction angle of 2θ.

### Evaluation of Infrared Spectrum (FT-IR)

**Figure 3:**
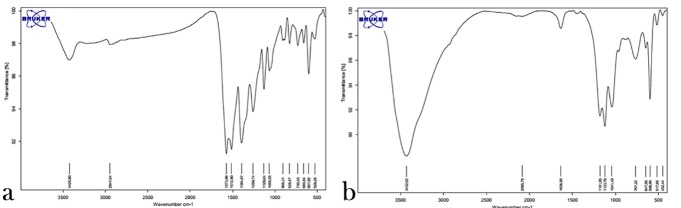
FT-IR images of Ag (a) and ZnO (b) nanoparticles.

The IR spectrum of silver nanoparticles (a) and zinc oxide nanoparticles (b) in Figure 3 shows their correct synthesis considering the absorbed bands of the reactive groups of each nanoparticle.

### Determination of Antifungal Properties (against C. albicans) of Tissue Conditioner Samples Containing 0.625, 1.25, 2.5, 5, 10 and 20 wt% of ZnO‒Ag Nanoparticles

Table 1 presents the antifungal properties of tissue conditioner samples containing different percentages of ZnO‒Ag nanoparticles at 24- and 48-hour intervals. The results of ANOVA showed that complete inhibition of *C. albicans* occurred at 24- and 48-hour intervals at 10% and 20% concentrations. In general, the best effects were achieved with 5% concentration of ZnO‒Ag at 24- and 48-hour intervals.

**Table 1:** the antifungal properties of tissue conditioner samples containing different percentages of ZnO,Ag nanoparticles at 24- and 48-hour intervals.

	*C. albicans*			
Concentration	24-hour		48-hour	
	mean	SD	mean	SD
0.63	1.52	0.20	1.04	0.17
1.25	1.35	0.27	0.93	0.21
2.5	1.10	0.15	0.78	0.19
5	0.65	0.34	0.31	0.17
10	0	0	0	0
20	0	0	0	0
control	10.43	5.20	14.27	3.17
P-value	0		0	

Table 2 presents the results of Tukey tests for two-by-two comparisons of ZnO‒Ag nanoparticles in relation to the antifungal properties. As indicated, an increase in the concentration of ZnO‒Ag nanoparticles resulted in significantly less turbidity compared to the control sample (P<0.05).

**Table 2:** the results of Tukey tests for two-by-two comparisons of ZnO,Ag nanoparticles in relation to the antifungal properties.

		***C. albicans***			
**Concentration (%)** **(I)**	**Concentration (%)** **(J)**	**24-hour**		**48-hour**	
		**(I-J)**	**P-value**	**(I-J)**	**P-value**
**control**	**0.63**	8.91	0	13.63	0
**0.63**	**1.25**	0.17	0.73	0.11	0.85
**1.25**	**2.5**	0.25	0.14	0.15	0.27
**2.5**	**5**	0.45	0.03	0.47	0.04
**5**	**10**	0.65	0	0.31	0.04
**10**	**20**	0	-	0	-

In addition, at both 24- and 48-hour interval, 10% and 20% concentrations of nanoparticles resulted in complete inhibition of the growth of microorganisms.

### Determination of the Antibacterial Effects of Tissue Conditioner Samples with 0.625, 1.25, 2.5, 5, 10 and 20 wt% of ZnO‒Ag Nanoparticles against S. mutans, E. faecalis and P. aeruginosa

Table 3 presents the antibacterial properties of tissue conditioner samples containing different concentrations of ZnO‒Ag nanoparticles at 24- and 48-hour intervals. The results of ANOVA showed the following:

**Table 3:** the antibacterial properties of tissue conditioner samples containing different concentrations of ZnO,Ag nanoparticles at 24- and 48-hour intervals.

	***S*** ***. mutans***				***E. faecalis***				***P. aeruginosa***			
**Concentration**	**24-hour**		**48-hour**		**24-hour**		**48-hour**		**24-hour**		**48-hour**	
	**mean**	**SD**	**mean**	**SD**	**mean**	**SD**	**mean**	**SD**	**mean**	**SD**	**mean**	**SD**
**0.63**	4.59	1.60	3.85	1.57	1.87	0.30	1.7	0.27	2.45	0.70	1.52	1.10
**1.25**	4.55	0.67	3.64	2.61	1.73	0.37	1.6	0.31	2.21	0.69	1.22	0.26
**2.5**	4.23	1.51	2.73	1.07	1.02	0.46	0.83	0.29	1.30	0.91	0.40	0.18
**5**	2.23	0.68	1.65	0.60	0.87	0.42	0.56	0.17	0.53	0.15	0.29	0.12
**10**	1.26	0.52	1.07	0.5	0	0	0	0	0	0	0	0
**20**	0	0	0	0	0	0	0	0	0	0	0	0
**control**	13.27	2.67	17.83	4.29	11.34	5.37	12.41	4.29	10.76	4.31	13.18	3.54
**P-value**	0		0		0		0		0		0	

In relation to *S. mutans*, complete inhibition of growth occurred at 24- and 48-hour intervals at 20% concentration. In general, the best antibacterial effects were achieved at 24- and 48-hour intervals with 10% concentration of ZnO‒Ag.

In relation to *E. faecalis* complete inhibition of growth occurred at 24- and 48-hour intervals at 10% and 20% concentrations. Generally, the best effects at both 24- and 48-hour intervals occurred with 2.5% concentration of ZnO‒Ag.

In relation to *P. aeruginosa*, complete inhibition of growth at both 24- and 48-hour intervals occurred at 10% concentration of nanoparticles. The best effects occurred at 24-hour internal with 5% and at 48-hour internal with 2.5% concentration of ZnO‒Ag.

Table 4 presents the results of Tukey tests for two-by-two comparisons of different concentrations of chitosan in relation to its antibacterial effects.

**Table 4:** the results of Tukey tests for two-by-two comparisons of different concentrations of chitosan in relation to its antibacterial effects.

		***S. mutans***				***E. faecalis***				***P. aeruginosa***			
**Concentration (%) (I)**	**Concentration (%) (J)**	**24-hour**		**48-hour**		**24-hour**		**48-hour**		**24-hour**		**48-hour**	
		**(I-J)**	**P-value**	**(I-J)**	**P-value**	**(I-J)**	**P-value**	**(I-J)**	**P-value**	**(I-J)**	**P-value**	**(I-J)**	**P-value**
**control**	**0.63**	8.86	0	13.98	0	9.47	0	10.71	0	8.31	0	11.66	0
**0.63**	**1.25**	0.04	0.74	0.21	0.45	0.14	0.73	0.10	0.45	0.24	0.35	0.3	0.15
**1.25**	**2.5**	0.32	0.05	0.91	0.06	0.71	0.04	0.77	0.03	0.91	0.04	0.82	0.04
**2.5**	**5**	2.00	0	1.08	0	0.15	0.37	0.27	0.28	0.77	0.04	0.11	0.25
**5**	**10**	0.87	0.02	0.58	0.03	0.87	0.03	0.56	0.04	0.53	0.12	0.29	0.45
**10**	**20**	1.26	0.03	1.07	0.03	0	-	0	-	-	-	-	-

With all the three bacterial species, an increase in the concentration of ZnO‒Ag nanoparticles resulted in significantly less turbidity compared to the control samples.

In relation to *S. mutans*, 20% concentration of nanoparticles resulted in complete inhibition of bacterial growth at 24- and 48-hour intervals. In relation to *E. faecalis* and *P. aeruginosa*, 10% and 20% concentrations of nanoparticles resulted in complete inhibition of bacterial growth at 24- and 48-hour intervals.

## Discussion

With the advent of nanotechnology, silver nanoparticles were produced with strong antimicrobial properties.^24,25^ Silver nanoparticles have exhibited unique interactions with different bacterial and fungal species^24,26^ and are used in different dental fields, including endodontics,^27,28^ prosthodontics (dentures) and implantology^29,30^ and restorative dentistry.^31,32^ The aim of the use of silver nanoparticles is to minimize microbial activity in dental materials, promote orodental health and improve the quality of life. In addition, ZnO nanoparticles have antimicrobial effects and have advantages over silver nanoparticles, including lower cost, a white appearance and the ability to block ultraviolet light.^33^

In the present study, incorporation of ZnO‒Ag nanoparticles into tissue conditioner samples resulted in a decrease in the growth and proliferation of *C. albicans*. In this context, 0.625 wt% of these nanoparticles resulted in a significant decrease in the growth of *C. albicans* compared to the control samples at both 24- and 48-hour intervals. Complete inhibition of growth occurred with 19% concentration at both 24- and 48-hour intervals. Generally, the best growth inhibition at both 24- and 48-hour intervals occurred at 5% concentration of ZnO‒Ag.

Nam et al showed that incorporation of 0.5 wt% of silver nanoparticles into a tissue conditioner (GC soft-liner) resulted in complete inhibition of *C. albicans* colonization.^18^ Since Nam et al used only silver nanoparticles, which have a high antibacterial effect, the complete inhibition of growth occurred at lower concentrations.

In a study by Li et al, 1‒5 wt% of silver nanoparticles in acrylic resin exhibited antifungal activity.^19^ In the present study, too, 5 wt% of ZnO‒Ag nanoparticles exhibited a significant effect on decreasing *C. albicans* colony counts.

Discrepancies in the results of different studies are attributed to differences in the materials tested (pure nanoparticles or a combination of several nanoparticles), differences in the culture media, differences in bacterial serotypes and the technique used to determine bacterial growth (turbidity, colony counts, etc.), which should be taken into account in studies and evaluations.

In the present study incorporation of ZnO‒Ag nanoparticles into tissue conditioner samples resulted in a decrease in the growth of *S. mutans*. In this context, incorporation of 0.625 wt% of nanoparticles resulted in a significant decrease in the growth of *S. mutans* compared to the control samples at both 24- and 48-hour intervals. In relation to *S. mutans*, complete inhibition of growth occurred at 24- and 48-hour intervals at 20%, concentration. Generally, the best concentration of ZnO‒Ag nanoparticles was 10 wt% at both 24- and 48-hour intervals.

The bacterial growth curve consists of 4 phases, including delayed, increasing, stationary and death phases. Therefore, at lower percentages of nanoparticles (such as silver and zinc oxide) bacterial growth is not 100% and the bacteria continue to grow and proliferate. This indicates a lack of the effect at lower concentrations and the efficacy of nanoparticles at higher concentrations.^24^

Since metallic nanoparticles affect polymerization of dental materials at high weight percentages and result in a decrease in their biocompatibility, the minimum concentrations that are effective should be used.

Kasraei et al showed the antibacterial properties of composite resins containing silver and zinc oxide nanoparticles on *Streptococci*. They reported that the effect of zinc oxide on *S. mutans* was significantly higher than that of silver.^34^ Padmavathy et al and Sirelkhatim et al evaluated the properties of zinc oxide nanoparticles and reported it as an antibacterial agent which is biocompatible with human cells.^35,36^

Silver nanoparticles attach to cell membrane and also penetrate into the bacterial cell. The bacterial membrane has proteins containing sulfur groups and silver nanoparticles react not only with these proteins but also with components containing phosphorus such as DNA. In addition, these nanoparticles attach to respiratory chain (which is effective in cell division) and result in cell death. In addition, these nanoparticles release silver ions which in turn increase their antibacterial activity.^37^

In the present study, incorporation off ZnO‒Ag nanoparticles into tissue conditioner samples resulted in a decrease in *E. faecalis* and *P. aeruginosa* counts; in this context, incorporation of 0.625 wt% of nanoparticles resulted in a significant decrease in the growth of both bacterial species compared to the control samples at both 24- and 48-hour intervals. Complete inhibition of growth was observed at 24- and 48-hour intervals at 10% concentration. Generally, in relation to *E. faecalis* the best concentration at both 24- and 48-hour intervals was 2.5 wt% of ZnO‒Ag nanoparticles. However, in relation to *P. aeruginosa* the best concentration at 24-hour interval was 5%, with 2.5 wt% of ZnO‒Ag nanoparticles at 48-hour intervals.

The results of a study by Mahross and Baaroudi showed that 5% nanoparticles of silver had significantly highest mean storage modulus E' and loss tangent Tan δ values followed by 2% Ag nanoparticles (P<0.05). They suggested that incorporation of Ag NP into the acrylic resin denture base material can improve its viscoelastic properties.^38^

In addition, Srivastava et al showed that nanoparticles can be modified to improve the thermal and mechanical properties of various acrylic denture base resin materials.^39^ Moreover, further studies are needed to investigate long-term properties of nanoparticles incorporated into tissue conditioners.

## Conclusion

Incorporation of ZnO‒Ag nanoparticles into tissue conditioners resulted in the inhibition of bacterial proliferation.

## Recommendations

It is recommended that evaluation of antibacterial and antifungal properties of ZnO‒Ag nanoparticles for complete growth and effective concentration be carried out at a wider range so that the least concentration required can be more accurately determined.

## Competing interests

The authors declare no competing interests with regards to the authorship and/or publication of this article.

## Authors’ contributions

AK prepare proposals, set and enter the results of the studies and their interpretation, Prepare and interpret data, Prepare a final report, prepare results, writing the article AM supervised the design and execution of the study and Preparing a final report. NA collected the data and contributed to preparation of the proposal.

## Acknowledgments

The authors thank the Medical Microbiology and Nanotechnology Department of Tabriz University of Medical Sciences.
